# Asthma and Its Treatment

**Published:** 1909-04-10

**Authors:** Charles J. Whitby


### The General Practitioner's Column.

[Contributions to this Column are invited, and if accepted will be paid for.]

### ASTHMA AND ITS TREATMENT.

By CHARLES J. WHITBY, M.D.Cantab.

Noteworthy among the wise saws which most of us imbibed in our student days is one to the effect that asthma is "not a disease but a symptom." It is neither more nor less true than would be the statement that epilepsy, migraine, or any other of the paroxysmal neuroses is a "symptom." Asthma *may* of course be a symptom: I remember a well-marked case which was cured by the removal of nasal polypi, and no doubt this is common enough. But true asthma is as definite a pathological entity as cancer or chicken-pox. It has fallen to my lot to see a good many cases of this terrible malady—for terrible it often can be—and I have been struck by the fact that many of these have escaped recognition by the doctors who had previously seen them. Has this been a result of the fallacious teaching referred to above, I wonder? There is distinct evidence that true asthma is commonly of hereditary origin, the worst cases being those in which the disease declares itself in childhood and continues throughout life. The least disturbance of the ordinary routine of life, in regard to climate, diet, or exertion, will in such cases inevitably precipitate an attack, by which, for a week or fortnight, the patient will be absolutely incapacitated. Life may become a burden, and, failing relief or even recognition of their malady from the profession, such patients fall ready victims to the omnipresent quack and his vaunted specifics.

As to the pathology of the condition, the fundamental fact about asthmatics, who come as a rule of neurotic or otherwise impaired stock, is a congenital hyperæsthesia of the skin and mucous membranes, resulting in a tendency to reflex vasomotor and visceral disturbances. Asthmatics are peculiarly sensitive to smells: not only to bad smells, for even perfumes, which to others are delightful, often cause more or less dyspnœa. Frequently they suffer from hay-fever in the summer months. From the loud dry wheezing audible during both inspiration and expiration all over the chest, I infer a general spasmodic contraction of the bronchioles. Associated with this there is probably considerable engorgement of the mucosa and submucosa, as the attack invariably terminates in hypersecretion of mucus. The reality of the obstruction, *felt* more especially with regard to expiration, is proved by the fact that subcutaneous emphysema may (as in whooping-cough) be thereby induced. I once attended a young man who, in the course of a violent attack developed pronounced emphysema of the trunk, neck, and face. So far as my observations go, the arterial blood-pressure would seem to be invariably high during an attack of asthma, averaging perhaps 180 to 200 mm., the result, mainly, of spasm of the cutaneous arterioles. One must not, of course, overlook the probability of a toxæmic element in the production of asthma, so-called gouty asthma being an evident case in point.

Of the preventive treatment, the most important factor is the choice of a suitable climate. In my experience most asthmatic subjects have seemed to require a climate at once fairly bracing, yet sheltered from cold winds. Seaside places are only suitable during the summer months, and only then such comparatively mild resorts as Bournemouth, Dawlish, Weymouth, Sidmouth, etc. Places near the coast, yet sheltered from the direct inland winds by a range of hills (*e.g.* by the South Downs), are often found beneficial. And of course it is proverbial that many asthmatics are comparatively well in London. Perhaps the sulphurous contamination of the atmosphere has a medicinal effect! In regard to climate, and all else indeed, idiosyncrasy counts for much. I have heard of a husband and wife compelled by asthma to live respectively at the top and bottom of a hill.

It is of the first importance for these unfortunates to make a careful study of diet: an attack of dyspepsia is invariably the precursor of their enemy's appearance. They should, in most cases, avoid pastry and cheese, pork, pickles, all highly-pungent condiments—in fact everything of a chemically or mechanically irritant nature, and they should avoid *every* kind of excess. In the matter of apparel, asthmatics largely affect thick woollen underwear; "chest-protectors" are also a favourite vanity of their kind. Such things aggravate the cutaneous hyperæsthesia; they should be replaced by cellular material or by cotton combination garments. A woollen shirt may be necessary in cold weather, but it should not be worn next the skin. As supplementary hardening agents, a morning cool sponge bath or a graduated rose-douche, cold sponging of the chest, paddling, barefoot walking, and "air baths" may be recommended.

Systematic breathing exercises are of great utility as a preventive of asthma, sufferers from which are as a rule bad breathers. Debilitated patients may take them sitting or recumbent: stronger individuals should be taught a few carefully-considered exercises adapted for chest-expansion, and instructed to go through these a certain number of times after the morning bath. Directions cannot be too precise: vague recommendations of "exercises" without specific details are useless.

The first question one asks oneself when called to a patient in the throes of a bad attack of asthma is apt to be "Does he (or she) require an injection of morphine?" It is a question which, when in doubt, one should answer in the negative. Temporary relief can almost certainly be given, but one has to remember that one is dealing with a condition which may recur many a time. The patient will not forget the relief once obtained by morphine, and the temptation to demand a repetition will be overwhelming. On the other hand, alarming as the symptoms of a really bad attack may well appear to the novice, one knows that danger is practically nil, and one may generally hope to give relief by less questionable means. Still, in a small minority of cases, and in exceptionally severe attacks, an occasional injection of morphine and atropine is, I believe, good treatment. A patient may be so exhausted as hardly to realise what is being done to him. From first to last, during a genuine attack, the patient should be kept in bed in a warm but well-ventilated room. Until free expectoration has begun (the almost invariable precursor of recovery), the least exertion will certainly aggravate whatever amount of dyspnœa may exist. With regard to dietary—patients going through a bout of genuine asthma have an instinctive loathing for food. Digestion is in abeyance, for the vital powers are concentrated on the mere labour of respiration. The less food taken, the sooner, I believe, will the attack be over. An occasional cup of "Benger's" made partly with water, varied once or twice in the 24 hours by a cup of weak "invalid" (*i.e.* unseasoned) "Bovril," is as much as can be assimilated. Some relief often follows immersion of the hands and forearms for 10 to 15 minutes in very hot water. A foot-bath may be given simultaneously if the patient can be got out of bed.

Finally, as to drug-treatment: the main indications are (1) reduction of blood-pressure, if excessive, (2) promotion of expectoration, and (3) mitigation by sedatives of the spasm of the bronchioles. One or more of these desiderata is fulfilled by each of the following, all of which I have found helpful, alone or in combination: iodide of potassium (or any of the modern iodine preparations), bicarbonate of potassium, sodium nitrite, spirit of nitrous ether, vinum antimoniale, ethereal tincture of lobelia, fluid extract of hyoscyamus, fluid extract of valerian, morphine, heroin, codeine. When, as often, pungent or nauseous mixtures disagree, small hourly doses of lobelia or of antimony may cut short the attack. The inhalation of the fumes of powders containing stramonium, etc., is a useful palliative, but has no curative effect.

